# Carbon Nanotubes Filled with Ferromagnetic Materials

**DOI:** 10.3390/ma3084387

**Published:** 2010-08-13

**Authors:** Uhland Weissker, Silke Hampel, Albrecht Leonhardt, Bernd Büchner

**Affiliations:** Leibniz Institute for Solid State and Materials Research (IFW) Dresden, Helmholtzstrasse 20, D-01069 Dresden, Germany; E-Mail: s.hampel@ifw-dresden.de (S.H.); a.leonhardt@ifw-dresden.de (A.L.); b.buechner@ifw-dresden.de (B.B.)

**Keywords:** carbon nanotubes, ferromagnetic nanowires, growth mechanism, magnetic properties, application

## Abstract

Carbon nanotubes (CNT) filled with ferromagnetic metals like iron, cobalt or nickel are new and very interesting nanostructured materials with a number of unique properties. In this paper we give an overview about different chemical vapor deposition (CVD) methods for their synthesis and discuss the influence of selected growth parameters. In addition we evaluate possible growth mechanisms involved in their formation. Moreover we show their identified structural and magnetic properties. On the basis of these properties we present different application possibilities. Some selected examples reveal the high potential of these materials in the field of medicine and nanotechnology.

## 1. Introduction

Since their detection in 1991 by Iijima *et al.* [1] intensive studies have been conducted on carbon nanotubes. The unique structure of single walled carbon nanotubes (SWCNT) can be described as rolled-up graphene planes and their ends are closed by fullerene-like caps [2]. Multi-walled carbon nanotubes (MWCNT) can be considered as a stack of SWCNT, each having a different diameter that are inserted into one another [1].

Diverse methods can be applied for nanotube synthesis, the most popular being arc discharge [3], laser ablation [4], and chemical vapor deposition (CVD)[5]. Comprehensive reviews about this methods were previously presented by Moisala *et al.* [6], Melechko *et al.* [7] and Leonhardt *et al.* [8].

The exceptional electrical and mechanical properties of these sp${}^{2}$-hybridized molecular nanostructures make them one of the most promising building blocks for nanoscale science and nanotechnology. Their small size, large aspect ratio, ballistic transport properties and exceptional high Young’s modulus [9] render CNT promising candidates for applications in nanoelectronics, nanomechanics and composite materials [10]. In addition to these properties the CNT have chemically highly stable carbon shells and they can be opened and filled without losing their stability [11]. They can be filled with metals, semiconductors, salts, organic materials, therapeutics, *etc.*, either during the synthesis process (Fe, Co, Ni) [8,12,13] or through subsequent opening, filling and closing of the CNT [14,15]. The crystalline carbon shells protect the filling material from oxidation, the environment or against each other. So, they are qualified to be smart and safe carrier systems in the nanometer scale which can be filled with tailored materials to address specific purposes. Additionally, the CNT can be biofunctionalized [16,17,18,19] and provide a broad area of possible applications especially in biomedicine [15,19,20,21,22,23]. Especially, it has already been proven that CNT can penetrate into various cell types through biological barriers [24,25,26]. So far several studies report on drug loaded CNT as potential drug delivery systems [27,28,29,30,31].

In this article, we examine the state-of-the-art in the synthesis of CNT filled with ferromagnetic metals like Fe, Co, or Ni. After introducing some basic properties of CNT we discuss their characteristic magnetic properties in detail and highlight the opportunities and challenges to use them for potential applications, focusing on biomedical and sensor applications.

## 2. Synthesis of Carbon Nanotubes

**Figure 1:**
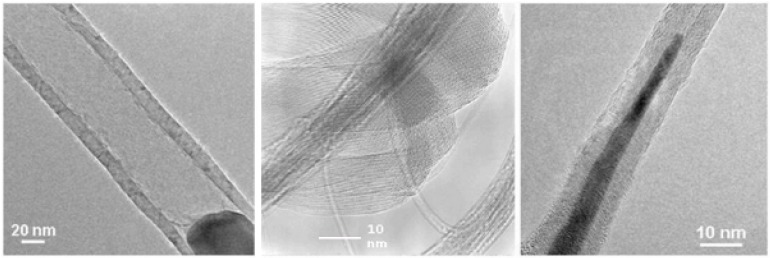
The image presents an empty CNT with spherical catalyst (left) bundles of SWCNT (middle) partially Fe-filled MWCNT (right).

The CVD is a convenient method for the synthesis of different types of carbon nanotubes ranging from the single-walled over the multi-walled to the metal-filled carbon nanotubes (see Figure 1). The method is suitable to produce large quantities with satisfactory quality and allows also an up scaling at moderate costs for industrial mass production. In this paper we focus on the manufacturing of filled carbon nanotubes. Based on the overview given by Leonhardt *et al.* [8] we want to show the progress in the synthesis of such tubes with defined properties as filling degree, length, diameter and form.

Basically the methods for the synthesis of empty and filled carbon nanotubes are similar. At both processes hydrocarbon precursor compounds are required which deliver the carbon for the formation of the CNT-walls. Additional, since it is a catalytic process, a catalyst (in this case a metal) is involved to control the kinetics of reactions such as the decomposition of the precursor. It also acts as a geometric confinement for the forming CNT-structure. The catalyst can be located on a substrate or is delivered via the gas phase. The latter is often called a floating catalyst process. While for empty CNT beside the metal-organic compound an additional hydrocarbon (mostly liquid or gaseous) is very often used, the deposition of highly filled carbon nanotubes is realized by only metal-organic compounds. The family of metallocenes (Me-(C${}_{5}$H${}_{5}$)${}_{2}$ with Me = transition metals) accommodates both elements (C and Me) in their structure in a fixed ratio of 1:10. Because these compounds also possess a convenient temperature range for sublimation and thermal decomposition, the metallocenes are the usually used precursors for filled carbon nanotube synthesis. Especially the metallocenes ferrocene, cobaltocene and nickelocene contain the transition metals Fe, Co and Ni respectively. It is well known these metals have a low carbon solubility, form only metastable carbides and are so able to act as efficient catalyst.

By using only such metallocenes the filling and the forming of the carbon nanotube is a simultaneous process (the so-called in-situ filling process). Other strategies, e.g., for an ex-situ filling of nanotubes in a separate step after the growth of empty, hollow CNT are not reconsidered within this review and are reported elsewhere [32,33].

In the following we focus on the in-situ filling processes and discuss two different methods, which were mainly developed in our work group. The first method starts with a metallocene powder as precursor and in the second method the metallocene is dissolved in a liquid hydrocarbon at the outset of the synthesis process.

### 2.1. Solid source chemical vapor deposition (SSCVD)

**Figure 2:**
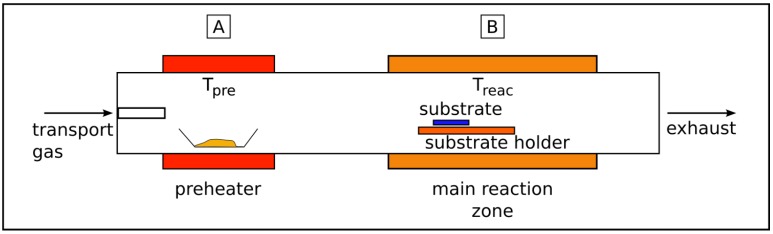
Setup of a typical SSCVD experiment. Ferrocene is sublimated in the preheater zone A at temperature T${}_{pre}$. The decomposition of the precursor and the formation of CNT occurs in the reaction zone B at temperature T${}_{reac}$.

For the solid source CVD a horizontal quartz tube and a two zone furnace can be used. The typical setup for the SSCVD method is shown in Figure 2. In the first temperature zone (T${}_{pre}$ ≪ T${}_{reac}$) the metallocene powder is positioned in a quartz boat and is directly sublimated at a compound specific temperature (e.g., for ferrocene in the temperature range between 100 and 200 *°*C) [34,35]. The metallocene vapor is transferred by a controlled argon, hydrogen or nitrogen flow (or mixtures of them) into the second temperature zone (T${}_{reac}$) where the decomposition of the metallocene and the growth of the filled carbon nanotubes occurs.

The chosen sublimation temperature (T${}_{pre}$) determines the sublimation rate and together with the rate of argon the amount of metallocene which is transported into the reaction zone. Thus the metallocene concentration is a function of temperature, gas flow and furnace geometry.

As it will be discussed in more detail later, the precursor concentration is a decisive factor for the properties of the obtained material. For the deposition of iron-filled carbon nanotubes the temperature T${}_{reac}$ is preferentially varied between 1023 and 1372 K. The deposition occurs on special substrates located in the hot zone of the reactor as well as on the inner wall of the quartz tube reactor. However, the reactor wall is more provided with undefined material compared to the aligned grown nanotubes on the substrate. Commonly, silicon substrates (semiconductor wafers) with a thin oxide layer are employed and an additional coating of these substrates with few nanometer thin metal layer (the metal could be Fe, Co or Ni) lead to an improved deposition behavior and higher filling degrees, because these layers act as secondary catalysts.

### 2.2. Liquid source chemical vapor deposition (LSCVD)

**Figure 3:**
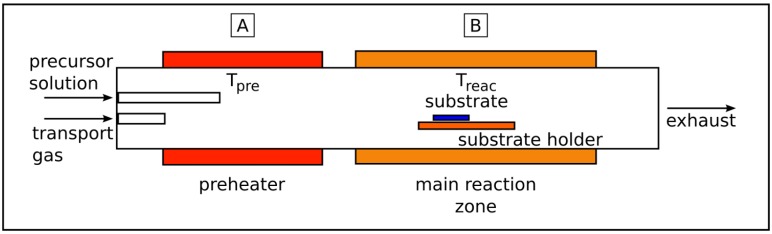
Setup of an aerosol experiment. The precursor solution is introduced by a nozzle and diluted by a transport gas flow. The solution evaporates in the preheater zone at temperature T${}_{pre}$. In the reaction zones both the hydrocarbon and the metallocene decompose at temperature T${}_{reac}$ to form the CNT.

The term “liquid source chemical vapor deposition” covers all methods employing solely liquid precursors as starting material for the deposition process. Because of its high flexibility and adaptability, the LSCVD method is widely used in the synthesis of various empty and filled carbon nanotubes [12,36,37,38,39]. The liquid precursor is either the only carbon source or it also acts as a solvent for other precursor compounds. In the case of carbon nanotube synthesis the liquid precursor is a hydrocarbon (benzene, acetonitrile, heptane, *etc.*) in which the metal catalyst compounds (very often metallocenes) are solved. If such liquids are introduced in the CVD reactor both the hydrocarbon and the metallocene decompose simultaneously. Caused by the high excess of carbon in the gaseous phase predominantly hollow carbon nanotubes with small spherical catalyst particles are formed but no filled CNT with long wires inside the tubes. The simplest experimental design for the LSCVD is a two zone furnace (horizontal or vertical) with an evaporation zone followed by reaction range. The principle setup is presented in Figure 3.

The injection of the liquid precursor into the reactor can be realized in different manner, but often by means of an additional inert gas flow such as argon or nitrogen. Actually, in the hot zone the decomposition of all precursor components takes place and the carbon nanotubes are formed. The attraction of the LSCVD method is the ability to control precisely the concentration of the used solution (the atom ratio between carbon and the metal component (catalyst) can be exactly regulated in a certain range (carbon/metal ratio ≫ 10). Furthermore, the feeding amount can be adjusted using a flux pump (e.g., syringe pump) [36,38,40,41]. A special type of the injection method is the spray pyrolysis of the precursor solution by utilizing an aerosol generator, which can be a nozzle or an ultrasonic bath, that produce the aerosol [37,40,42,43,44].

**Figure 4:**
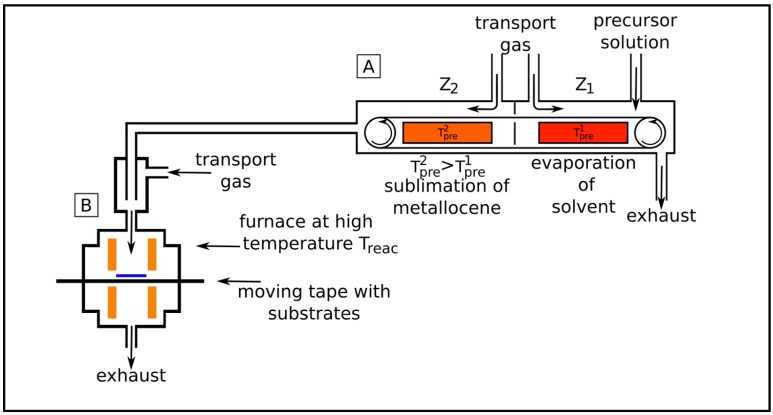
Setup of a LSCVD experiment. The precursor is prepared in device A. The solvent is evaporated at T${}_{pre}^{1}$ in zone Z${}_{1}$ and the ferrocene is sublimated at T${}_{pre}^{2}$ in zone Z${}_{2}$. The ferrocene mass flow is diluted by additional transport gas flow. In device B the reaction takes place at the temperature T${}_{reac}$. A moving tape allows for the continuous deposition of CNT on substrates.

Another LSCVD method for the synthesis of multi-walled carbon nanotubes with high filling degree is designed in our laboratory [12]. The setup is constructed for a continuous processing of mass-produced materials and is shown in Figure 4.

It consists of a continuously acting band evaporator and a hot wall reactor equipped with a movable tape as substrate holder. The liquid precursor (e.g., a solution of ferrocene in cyclopentane) can be dropped on the moving band of the evaporator with different speed. At constant band velocity the drop rate defines the amount of ferrocene which is transported into the reactor. The band evaporator is separated in two independent heating zones (Z${}_{1}$ and Z${}_{2}$ in Figure 4). In the first zone (Z${}_{1}$) the solvent (cyclopentane) evaporates at the temperature T${}_{pre}^{1}$ and is taken away by an argon flow directed to the exhaust. Due to the elimination of the solvent again a C to Me ratio of 10:1 could be achieved. In the second zone (Z${}_{2}$) the actual precursor (solid ferrocene) completely sublimates (evaporates) at the temperature T${}_{pre}^{2}$ and is transferred into the hot wall reactor supported by an additional inert gas flow (Ar). In the reactor the precursor decomposes above 800 *°*C and filled nanotubes are deposited on catalyst precoated substrates. It is substantial for the described method, that the boiling point of the solvent is much lower than the sublimation temperature of the metallocene. This is the only way to ensure a complete evaporation of the solvent without loss of metallocene. Also, very long deposition experiments can be performed depending only on the amount of the used solution. The method produces strongly aligned multi-walled carbon nanotubes with a high filling degree.

### 2.3. Application-oriented demands on filled carbon nanotubes

**Figure 5:**
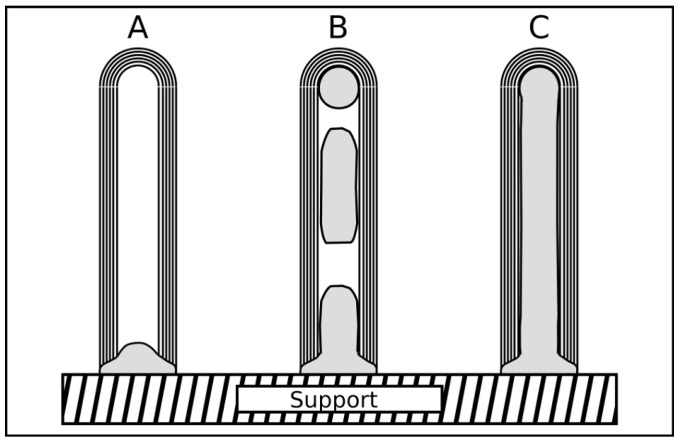
The sketch represents three types of filled CNT that possess different degree and distribution of the filling. All types are found in literature, however a long and continuous filling (C) is reported for iron by, e.g., [12,35,41,47,48], cobalt [13,49] and nickel [33,50]. Type A and B are especially interesting for applications where the cavity shall be filled with foreign material.

As already mentioned above, filled carbon nanotubes have an interesting application potential in different fields. But in dependence on the kind of application filled carbon nanotubes with tailored and tuned properties are required. For example, in the biomedicine a complete filling (compare Figure 5) is often not desired, rather a partially filled tube, because in this field the nanotube is used as multifunctional nanocontainer and the ferromagnetic filling is only one component inside the tube. On the other side, a continuous filling is stringently required for using the tubes as probes for magnetic force microscopy (MFM). A high aspect ratio of the filling leads to a geometrically extended magnetic dipole [45,46].

### 2.4. Discussion of synthesis parameters

In this section we will discuss the state of the art concerning the so far reached filling degree (and distribution) in dependence on the deposition conditions. Beside the filling degree, other very important properties of filled carbon nanotubes are their diameter and length, respectively. Both also determine significantly the application possibilities. The so-called aspect ratio, *i.e.*, length to diameter ratio strongly influences the physical and chemical properties of the filled nanotubes. Furthermore, another often demand is that the tubes are grown straight and not wool-like entangled and bent. Straight grown nanotubes can be better handled and show stronger anisotropic properties, which are very important for applications, such as utilizing the magnetic properties of the encapsulated nanowires or in composite materials. In this connection an alike important criterion for the quality of all types of nanotubes is the crystallinity of the carbon shells, because a lot of properties depend on its perfection.

Therefore in the following we want to show the connection between these properties (filling degree, length, diameter, morphology and shell structure) and specific used process parameters. They are mutually dependent and complement one another.

First the role of the precursor will be discussed followed by a description of the influence of different substrates, including additional interlayers and catalyst layers on the deposition process. Finally the effect of the reaction temperature and the gas atmosphere is presented. A critical analysis of the literature will give an overview about the current possibilities to produce filled carbon nanotubes with application-tuned properties.

**Figure 6:**
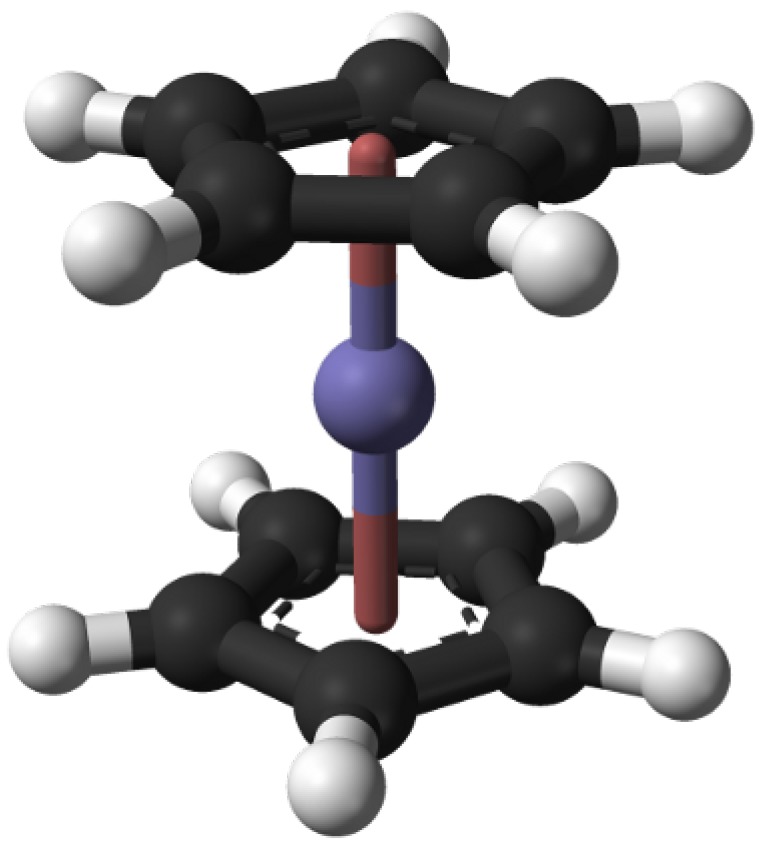
Ferrocene molecule as a representative of the general structure of metallocenes. Two cyclic organic molecules (ligands) bind to the central metal atom. In the center is the iron atom (blue), the carbon atoms are black and hydrogen atoms are white respectively.

The choice of appropriate precursors is important for a successful synthesis for both empty and filled carbon nanotubes. Fillings with ferromagnetic properties at room temperature are restricted to the elements iron, cobalt and nickel. The organometallic family of metallocenes, especially ferrocene, cobaltocene and nickelocene contain these metals in the molecule and therefore they are suitable as precursor for the synthesis of metal-filled CNT. Apart from their decomposition temperature also their kinetics and the reactivity of reaction by-products are convenient for a successful synthesis process [34,51]. A further advantage is that metallocenes can be combined. For example CNT with iron/cobalt alloys inside were obtained by mixing ferrocene and cobaltocene powders [52] or by dissolving both compounds in toluene [53]. The metallocenes are solid crystalline powders at room temperature. The structure is sandwich-like with the metal in the center and two cyclopentadiene rings as ligands. An example is shown in Figure 6. The material can be sublimated in a wide temperature range starting from 100 *°*C up to 300 *°*C. The sublimation behavior especially for ferrocene was intensively studied [54,55,56]. For the synthesis often a value of 150 *°*C is given as an optimum temperature.

The concentration of the metallocene in the gas phase at constant transport gas flow can be controlled by setting the sublimation temperature. Setting a higher sublimation temperature the concentration of the metallocene in the gas phase can be raised keeping the transport gas flow constant. An increase of the inner and outer diameter of iron-filled CNT was observed when the ferrocene concentration was hiked up. The increment of the outer diameter occurred since more carbon shells were formed by larger particles. At higher temperatures the metallocenes decompose according to the equation (1)

(1) $$Me{\left(C_{5}H_{5}\right)}_{2}\left(g\right)\to Me\left(s\right)+H_{2}+CH_{4}+C_{5}H_{6}+...$$

whereas the moieties depend on the reaction temperature and the composition of the gas atmosphere. The thermal stability of different metallocenes decreases from ferrocene over cobaltocene to nickelocene. Ferrocene decomposes above 1073 K whereas the nickelocene already above 550 K. In contrast, Dyagileva *et al.* [34] found a significant decomposition rate of ferrocene already at 550 *°*C in a hydrogen-containing medium. Since hydrogen is formed during the CNT growth the decomposition starts at 550 *°*C and a further rise of the reaction temperature will increase the decomposition rate and alter the composition of moieties in the gas phase.

Metallocenes can also be dissolved in liquid hydrocarbons as applied in the LSCVD method. The maximum solubility of ferrocene in selected solvents is given in Table 1. The higher the solubility of ferrocene in the solvent is the higher is also the possible concentration of metal in the reaction room. But with the injection the solvent also evaporates and decomposes depending on its thermal stability. The chosen solvents (Table 1) decompose in a wide temperature range (of 500 up to 1100 *°*C) and thus during the synthesis process more and less intensively. That means, more active carbon is there in the reaction zone and the consequence is a lower filling degree. Finally only partially filled CNT are formed [36,38,57,58]. The encapsulated particles can show a large variety of shapes. The particles are spherical or form small cylinders with low aspect ratio. It should be noted that the combination of ferrocene with diverse hydrocarbons leads to many different morphologies.

In contrast by employing only ferrocene as precursor high filling degrees up to 50% per tube could be achieved [8]. It should be emphasized that a large fraction of the CNT is filled with long and continuous iron nanowires.

**Table 1:** The table lists the maximum solubility of ferrocene in some liquid hydrocarbons that are often used as precursor. These organic compounds take part in the formation process and increase the carbon to iron ratio above 10:1.

compound	max. solubility of ferrocene	boiling temperature in *°*C
	in mg/ml at 293 K	
Ethanol	2	78
1-Propanol	1	97
cyclopentane	71	49
n-hexane	36	69
cyclo hexane	56	81
benzene	222	80
toluene	160	111
xylene	146	138–144
1,2-dichlorobenzene	236	180

Wang *et al.* [41] used chlorinated hydrocarbons such as 1,2-dichlorobenzene as solvent for the ferrocene (Table 1 last row). The boiling temperature is 454 K and thus in the upper end of the sublimation range of ferrocene. Two further special features of the 1,2-dichlorobenzene are important. In the presence of hydrogen hydrochloric acid can be formed which can interact with the iron or with carbon atoms. It was suggested that the etching effect of chlorine reduces the number of carbon shells and causes CNT with thin walls and smaller outer diameters [41]. To understand the influence of the chlorine different chlorinated compounds were investigated [41,48,59]. The other point is the special thermal decomposition behavior of the chlorine-contained hydrocarbon, since thermal stable benzene might be formed and therefore less active carbon is supplied to the CNT formation. The kinetics of the decomposition also depends on temperature and the retention period of a gas volume element in the hot zone of the furnace. Thus the decomposition of stable aromatic systems is more reduced at high transport gas flow. This results in less active carbon which in turn leads to shorter or thinner CNT.

In contrary ferrocene can be dissolved in cyclopentane which evaporates at much lower temperatures. This can be used to dispense the ferrocene in the LSCVD since the hydrocarbon can be completely evaporated and removed before the reaction takes place. In the formation of iron-filled CNT no additional carbon from the evaporated solvent participates. Therefore a very high filling yield above 45 wt % was achieved by the LSCVD method [12].

By using ferrocene as the only precursor a high filling degree can be expected. Any addition of reactive hydrocarbons will lead to a decrease of the filling degree. Inferential, the ratio of reactive carbon to reactive iron in the gas phase is an important control parameter for the filling degree and filling distribution.

The constitution of the substrates is also a very influential factor in the synthesis of carbon nanotubes [60,61,62,63,64]. The substrate acts as support for the nucleation and growth of the CNT. The substrate should be thermally stable under process conditions and should also be inert for reactions with the different metal catalysts and with carbon. The most simple and inert substrate is the inner surface of the quartz tube reactor. Due to the floating catalyst conditions catalyst particles can condense at the surface and nucleation and growth of CNT occur. However, growth conditions can be optimized if special substrates are positioned at defined locations within the temperature profile of a furnace, because the growth conditions strongly depend on the location in the horizontal tube reactor [51,65]. Kuwana *et al.* [51] investigated the formation of small iron particles by ferrocene decomposition. Best conditions for small and homogeneous particles were found in the center line of the furnace.

Besides the position influence, substrates with defined preparations can alter the deposition process. The most common substrate types are silicon wafers with a thermal oxide layer on top. Besides quartz sapphire substrates are very often used. Both substrates show the required thermal stability and chemical inertness. Not only the chemical properties but also the physical interaction between substrate surface and metallic catalyst is important since, e.g., the size of a catalyst particle is a function of the surface tension of both substrate and catalyst and also the wetting behavior on the substrate.

The deposition of CNT on defined substrates has also the advantage that the substrate surface can relatively easily be changed by precoating with catalyst material and patterning the catalysts in order to obtain a better control of the deposition process. However, especially in the floating catalyst method it is difficult to prevent a growth on special parts of the substrate because both the carbon and catalyst are delivered via the gas phase. This is one issue that could be solved by employing additional interlayers. After the substrate has been chosen a decision about the interlayer has to be done.

**Figure 7:**
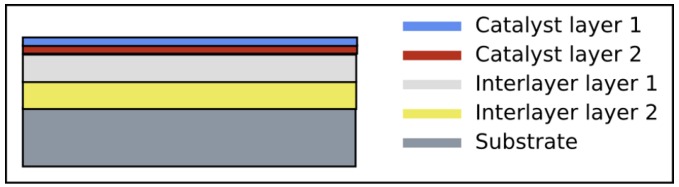
The substrate most often consists of a silicon wafer that is covered with a thin oxide layer. One or more interlayers such as alumina, tantalum, copper or tungsten can be employed. On top a thin layer of catalyst is deposited. If needed, the catalyst can be structured. For the formation of alloys more than one catalyst layer can be deposited. Multilayers also do enhance the activity of the catalyst.

As interlayers one or more thin layers of additional material that should adjust the properties of the substrate (e.g., silicon oxide) and the catalyst are employed. The basic structure of a substrate with interlayers is sketched in Figure 7. Like the substrates the interlayers also have to be thermally stable and chemically inert. Most often the interlayers are used to enhance the activity of the catalyst in order to achieve long and defect-free CNT. In some cases interlayers are used as inhibitor to prevent the formation of CNT at defined areas on the substrate.

The size of the catalyst particles is a decisive factor in the CVD to control the CNT diameter. The diameter distribution and the density of catalyst particles can be varied by the properties of the interlayer. The important feature is the wettability behavior between the interlayer and the catalyst material.

To enhance the growth of CNT the element aluminum is often used as interlayer material in the synthesis of CNT either as pure metal film [62,66,67] or as alumina [68,69,70]. It was found that these layers lead to a smaller mean diameter and diameter distribution of the CNT in the synthesis of unfilled as well as of filled CNT. The area density of CNT raised and the alignment of CNT increased. Furthermore chemical reactions of carbon on the interlayer surface might be involved [69]. The layer should be thicker than 1 nm in order to completely cover the substrate. A thickness of 10 nm alumina was reported to give high quality iron-filled CNT [71]. Consequently, such interlayer can change the catalyst particle size as well as its distribution on the substrate and thereby the diameter of the nanotubes [72,73,74].

Also other layers can be used as interlayers. Ng *et al.* [66] investigated the catalytic activity of Fe, Co, Ni and Fe/Ni in combination with several substrate materials such as Ti, Ta, W, Ir and Al. By using a combinatorial library it was found that most of the combinations allow for the growth of CNT. Except Al all materials have in common a very high melting point. In a carbon containing atmosphere Ti, Ta and W form stable carbides. The best result was obtained for Fe/Ni on Al. Iron gave good results on W and Al however, the best results were achieved on Ti. The Fe/Ni alloy showed the best results on all substrates. The combination of Ni and Al showed good results as well. For Fe and Co a higher density of CNT was found with Ti. In Table 2 the results are presented after [66].

**Table 2:** The table lists the synthesis results of the transition elements Fe,Co and Ni (as catalyst) on different metal interlayers. The classification into *best, good* and *bad* is related to the morphology of the obtained CNT. The CNT were grown on the different substrates under the same synthesis conditions. However, the results may differ to some extent if the growth conditions are optimized for each combination of interlayer and catalyst respectively [66].

catalyst	interlayer material		
	best growth conditions	good growth conditions	bad growth conditions
Fe	Ti	W, Al	Ta, Ir
Co	Ti	Ta, Al	W, Ir
Ni	Al	Ta, Ti	W, Ir

Another important feature of the interlayer is its possibility to act as an inhibitor. In special applications non-inert layers are deliberately used to locally prevent the growth of CNT [75]. This is important for the floating catalyst method especially if the material should only grow on selected areas. The growth of CNT based on ferrocene as precursor was successfully prevented by employing an amorphous carbon layer [76]. The iron from the gas phase falls onto the amorphous carbon and diffuses into the layer possibly forming carbides. It was also found that the iron diffuses through the amorphous carbon layer and accumulates at the interface between the silicon substrate and the amorphous carbon as small spherical iron-containing particles [76]. In principle elements that form very stable carbides can be imagined as potential inhibitor materials. Of course the suppression of the formation strongly depends on the interaction between the interlayer and the catalyst. In Table 2 the last column lists catalyst-interlayer configuration that result in very low quantity or no CNT formation [66]. These materials could be used as inhibitor materials in the floating catalyst method. Nevertheless, it remains a challenge to grow ferromagnetically-filled CNT in pattern.

To conclude, due to their influence on the catalyst particle density interlayers are used to control the density and alignment of CNT films. The interlayers should support the formation of small particles with a narrow diameter distribution and a homogeneous particle area density.

The most common catalyst materials are the VIIIb-metals of the periodic table of elements [66,77]. They have a low carbon solubility and form only meta-stable carbides. These are important properties of the catalyst material. However, an exclusive consideration of the influence of catalyst material on the CNT formation is somewhat artificial, since it is always in a strong interaction with the used substrate and interlayers respectively [60]. For the fabrication of well-filled carbon nanotubes two sources of catalyst material are available. The first is a metallocene in the floating gas and the second is a thin catalyst layer on the substrate. The size of the particles is a very important control parameter. As mentioned before the interaction of the catalyst material with the substrate surface is a basic issue in the catalyst preparation. Demands on the catalyst are the following: Defined diameter and small diameter distribution, low mobility on the surface thus low agglomeration, possibility to solve carbon, ability to form meta-stable carbides and to allow for surface or bulk diffusion.

At the nanoscale the catalyst affects the reaction not only by its chemistry but also by its geometry. The geometry of the catalyst in turn is affected by the interaction of the catalyst with its surrounding [60,61,63,64,77,78]. The so-called size effect, caused by the nanodimension of catalyst particles is intensively discussed in the literature [61,63,64,77,79,80,81,82,83,84]. Thereby it is possible to synthesize CNT at temperatures far below the melting point of the VIIIb-metals, because nanoparticles have lower melting points than the bulk material. A comprehensive overview about different methods for the preparation of catalyst was presented by Dupuis *et al.* [61].

A simple method for the production of catalyst particles is the deposition of thin films. A thermal treatment transforms the layers into small islands. In general, the growth of CNT by thermal CVD on a substrate is performed with catalyst precoated substrates. Such thin catalyst layers are sputtered or evaporated onto these substrates with thicknesses varying between 1 and 100 nm.

It is advantageous if the catalyst particles are formed by the Volmer-Weber mode (island mode). Islands are formed since the thin layer material wants to interact with itself rather than with the substrate or interlayer surface. In contrast, if the catalyst interacts corresponding to the Frank-van der Merve mode, which is the layer-by-layer mode, no separated particles would occur. In a third mode (called Stranski-Krastanov mode) the two before mentioned are combined. In this mode first a layer is formed but with a raising amount of material formation of islands starts. Therefore it is called the layer-plus-island mode. In the last situation also catalyst particles might be formed. In the synthesis of CNT by CVD most often the Volmer-Weber mode is assumed. However the last mentioned can not be excluded. As will be discussed in the section about the growth mechanism the interaction between the catalyst and the substrate determines the growth mode. In the Volmer-Weber mode the catalyst particles can have a contact angle below or above 90 *°*. In the first case there is a strong interaction between the particle and the surface and it broadens. In the second case the particle surface interaction is weak and it does not broad.

For the formation of small catalyst islands the catalyst should follow the Volmer-Weber growth mode. Therefore the interaction with the substrate is important and also the surface tension of the catalyst material. It is known, liquid Co forms smaller particles than Fe on a silicon oxide layer, because it has a higher surface tension [67]. A thin cobalt catalyst layer (2 nm) on SiO${}_{2}$ resulted in a visual decrease of the inner diameter in comparison to a 2 nm Fe layer. All other conditions were kept constant. It was suggested that a higher surface tension of the Co nanoparticles on the surface caused the smaller particle diameter. Thus the diameter of CNT can be controlled by the choice of the catalyst material, furthermore by the catalyst layer thickness and the pretreatment conditions.

The size of the catalyst particles defines the inner diameter of a CNT. The larger the particle diameter the larger the inner diameter is. To some extent the outer diameter depends on the catalyst particle size in a similar way; it increases with increasing particle size. However, the outer diameter also depends on other parameters, e.g., the reaction temperature and concentration of active carbon in the gas phase.

Besides all influences which are mentioned before, the particle size depends also on the temperature of the pretreatment of the substrate and the gas atmosphere. It was shown, that an annealing of thin catalyst films in the presence of hydrogen produces smaller particles than in a pure argon atmosphere [85]. The properties of catalyst particles are comparably important for the growth of both unfilled and filled CNT. At the deposition of unfilled CNT on prepared substrates the catalyst defines the growing site and the diameter of the CNT. However, before we discuss the influence of an additional catalyst layer on the growth behavior of filled CNT, we present some remarks about the synthesis of CNT by using the floating catalyst method. In this case the decomposition of the precursor in the gas phase leads to the formation of metal clusters. These clusters agglomerate on the substrate in a random manner and form the heterogeneous nucleation sites. The precursor concentration defines the average size of the particles.

**Figure 8:**
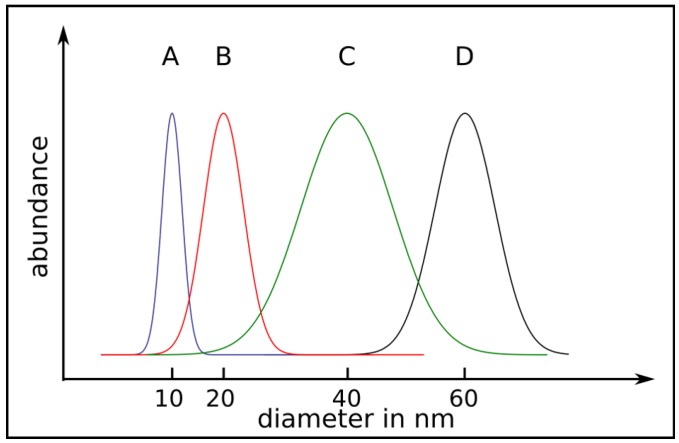
In the graph the mean outer diameter and the diameter distribution of CNT is sketched. Curve (A): small diameter and distribution of unfilled CNT grown on substrates with catalyst. The other curves (B,C,D) represent CNT grown by the floating catalyst method. Curve (B): almost unfilled CNT, Curve (C): well filled CNT, Curve (D): well filled CNT grown by the combination of catalyst supported substrates and floating catalyst. In principle the CNT possess a broader diameter distribution for the floating catalyst method. The given mean diameters are typical values for multi-walled CNT.

By comparison of floating catalyst experiments without and with additional catalyst layers on substrates the following correlation could be found. In the first case the average diameter of the CNT is smaller than in the second but the diameter distribution is much higher. Without additional catalyst it was found that the diameter strongly depends on the ferrocene concentration in the gas phase [8]. The principle correlation between the diameter and its distribution depending on the synthesis method is shown in Figure 8.

In the synthesis of metal-filled CNT by the LSCVD method there is often no additional catalyst supplied with the substrate. The filling degree is reduced not only because of the increased carbon to metal ratio but also due to the missing additional catalyst material on the substrate. However, substrates with additional catalyst can be used in the LSCVD as well [12,44].

In the presence of an additional catalyst layer the mean diameter of CNT increases, because in such case the size of the primary catalyst islands are dominantly determined by the thickness of the catalyst film. Thus, the diameter depends less on the ferrocene concentration [8]. In addition the alignment of the CNT grown by the SSCVD method enhances if there is catalyst on the substrate [8]. It was further revealed that the density of catalyst islands and the density of CNT corresponds [85].

The combination of both sources the catalyst material from the gas phase and from the substrate results in a high filling degree up to 50 wt% [8,12,47,67,86]. Without an additional catalyst layer a lower filling degree was found in the SSCVD method.

The catalyst is one of the most important parameter in the synthesis of CNT by CVD. Its activity does strongly depend on the synthesis conditions especially the reaction temperature and gas atmosphere. After the catalyst has been selected the optimal temperature and gas conditions must be found.

All reactions in the thermal CVD including sublimation, evaporation and decomposition of the precursors as well as the formation of CNT are strongly temperature dependent. The influence of the temperature and gas atmosphere on the formation of CNT are strongly interlinked. Intensive numerical simulation studies have shown [51,65,87,88,89], that the reaction temperature influences the catalyst particle formation and the kinetics of the gas phase reactions. It was found that the diameter of catalyst particles, which are spontaneously formed in the gas phase or on a substrate, increases with raising temperature. It is experimentally confirmed that the results significantly differ depending on the substrate position in the reactor. The reasons are the always existing temperature profiles in the reactor (radial and longitudinal) which depend on the utilized particular setup. These results confirm the sensitive dependence of CNT growth on the temperature and gas atmosphere. Besides the temperature profiles, each reactor has specific flow conditions which also strongly influence the deposition [51]. Because of that a comparison of the experimental results in the literature is difficult.

The sublimation and decomposition behavior of the widely used metallocenes were investigated in detail [34,54,56]. For example the decomposition of ferrocene in vacuum starts at temperatures ≈1,100 K, in contrast in a hydrogen flow already at temperatures of ≈700 K. However, there is a wide variation in the behavior within the group of metallocenes. The decomposition kinetics of hydrocarbons depends on the hydrogen concentration [34,56].

Here we want to discuss how the temperature and gas atmosphere influence the morphology of filled CNT grown by CVD. It should be emphasized that a change in temperature in order to control one particular parameter will always influence others as well due to the temperature dependency of the process parameters.

The morphology of CNT is characterized by the length, the outer and inner diameter and the shell thickness. In the case of (in-situ) filled CNT also the structure of the filling has to be considered. We present the influence of temperature and gas atmosphere onto these CNT properties.

The inner diameter and to some extent the outer diameter of a CNT are controlled by the diameter of the catalyst particle [73,74,90]. The diameter of the catalyst particles depends on the temperature but also on the gas atmosphere [85,91]. For example a pretreatment of 2 nm Fe films in an Ar/H${}_{2}$ atmosphere (30:1) at 1,100 K produces smaller islands in comparison with a treatment under pure Ar at the same temperature. In the first case the surface roughness was higher than in the second [85]. This is due to the different surface energy of the particles in the corresponding gas atmosphere. At high temperature catalyst particles on the substrate surface coagulate into larger islands. At lower temperatures the islands are smaller due to a lower activation energy and thus the surface mobility is hampered. Thus the pretreatment conditions (gas and temperature) are important for the diameter that can be expected for the CNT. However, due to the floating catalyst method an random raise of the particle diameters should be considered because of metal clusters from the gas phase that accumulate on the substrate surface.

In the floating catalyst method without additional catalyst on the substrate the formation of clusters and the rate of cluster deposition is related to the temperature and used gas phase. At low temperatures the decomposition is slow and only few metal cluster deposit on the substrate. However the decomposition rate of metallocenes increases in the presence of hydrogen and this would increase the deposition rate. Hence the balance between the temperature and the hydrogen concentration controls the diameter of the CNT.

So far we discussed how the temperature and the gas atmosphere act on the catalyst particle and thereby on the diameter. Now the direct influence of these two parameters onto the diameter, shell thickness and length will be investigated.

The most common inert or transport gas is argon, but also nitrogen [92] is used. Hydrogen containing gases such as pure hydrogen (H${}_{2}$) or ammonia (NH${}_{3}$) are often employed, but they cannot be considered as inert gas since they take actively part in the reaction. They are able to etch excess carbon away from catalyst particle surface [37]. Furthermore, the role of hydrogen is to limit or alleviate the so called poisoning of the catalyst particle. The catalyst particle can be deactivated if its complete surface is covered by excess carbon. By the loss of its catalytical activity the growth process will stop. The removal of excess carbon is crucial for a long active period of the catalyst and thus for the length of the CNT. If necessary, the growth of CNT can be stopped by a sudden increase of highly reactive hydrocarbons. If the diffusion of dissolved carbon through the particle is lower than the decomposition and deposition rate of carbon on the catalyst surface the growth will terminate. The growth process could also be finished by cooling which hinders the decomposition of hydrocarbons and diffusion of carbon.

Since hydrogen can have competitive effects on different occurring reactions its influence has to be carefully considered. The etching of the catalyst to maintain its activity is a desired effect. If the concentration of hydrogen is too high no CNT will form but oil-like deposits. Therefore the growth of CNT will also stop at a high concentration of hydrogen [93]. The role of hydrogen was discussed within a concept that explains its importance during the CNT formation by a so called free radical condensation mechanism [93].

Currently it is not completely clear whether such non-inert gases as hydrogen etch carbon from the deposited structure or if its influence is mainly on the gas phase reactions. Also the waiver of pure hydrogen can lead to a better growth behavior if only argon is used as transport gas as Müller *et al.* [35] could report. It is suggested that the amount of H${}_{2}$ produced by the ferrocene decomposition is sufficient. The total length of CNT did not increase with additional H${}_{2}$ but the filling degree decreased.

Another interesting observation during the growing of filled CNT is the sudden growth stop after a certain time [12], caused by the deactivation of the catalyst. Even though the CNT grown by LSCVD are in general much longer than those of the SSCVD, the reason is the same in both cases. The maximal observed length of CNT using the LSCVD is ≈45 *μ*m and only ≈15 *μ*m for SSCVD-technique. It was suggested that the continuous delivery of ferrocene (LSCVD) into the furnace allows for an uninterrupted growth since there are no strong oscillations in the precursor concentration. Nevertheless, the final length as well as the diameter are proportional to the ferrocene concentration [8]. However, it was proposed that the growth of very long CNT is due to the continuous feeding of iron catalyst that results from the ferrocene dissolved in xylene [38]. In that case the iron concentration was much lower in comparison to methods that solely employ ferrocene. There is no conclusive explanation for these experimental observation up to now.

It is difficult to grow short CNT by LSCVD (shorter 1 *μ*m) because of the high amount of active carbon. The high growth rates in LSCVD (≈1000 nm/s) are sometimes unfavorable since the length and diameter can hardly be controlled. Depending on the precursor the growth rate can be controlled by adjusting the ferrocene concentration, the gas composition and temperature. For example in the LSCVD (Figure 4) the diameter and length was smaller at a low ferrocene concentration but both are increased with raising concentration. The averaged growth rate which can be reached in the SSCVD method is in general lower (≈20–50 nm/s) than in the LSCVD. Therefore, this method is better suitable for the synthesis of shorter CNT.

The growth rate of a CNT, its diameter as well as the length depend on the decomposition rate of the precursor, which in turn strongly depends on the temperature and the gas atmosphere. Above a certain limit temperature the diameter of the growing CNT increases. This limit temperature depends also on the composition of the gas atmosphere [94]. It was found that the optimal temperature for CNT formation increases with increasing of the concentration of additional NH${}_{3}$ in the gas phase. For a higher temperature the precursor concentration should be increased. This is related to the balance between the precursor decomposition and the diffusion rate of dissolved carbon through the catalyst particle [94]. Wasel *et al*. [95] intensively studied the role of hydrogen in the formation of CNT. It was argued that with increasing hydrogen concentration the dehydrogenation of hydrocarbons is reduced and less active atomic carbon is available [93,95].

The outer diameter depends stronger on the reaction temperature than the inner diameter of the CNT, which is mainly defined by the catalyst particle diameter. At higher temperatures, the number of shells and also amorphous carbon deposition rate on the surface of already existing CNT increases. Juang *et al.* [94] found a significant increase of the diameter above 900 *°*C.

An increase of reaction temperature, e.g., above 1,000 *°*C did not lead to longer CNT but to an increase of the number of amorphous carbon coated particles especially in the synthesis of CNT from ferrocene precursor [8]. The wall thickness and thus the outer diameter is influenced by the reaction temperature. In principle the crystallinity of the carbon shells enhances with increasing temperature as confirmed by Raman spectroscopy [12]. Raman spectra of iron-filled CNT are shown in Figure 16.

It can be concluded that by a higher concentration of additional etching gases the growth temperature can be higher and still the wall thickness of a CNT can be reduced. In contrast, by using ferrocene the increased decomposition rate has to be considered, since this could lead to an increase of the diameter. If the carbon supply at the decomposition site of the particle is at an identical rate as the diffusion rate of carbon through the particle CNT will grow with best quality [94]. The total flow rate can be adjusted using a mixture of an inert gas (argon) and a reactive gas (hydrogen, ammonia) and for each compound used new optimal values for the parameters have to be determined.

Besides hydrogen the etching effect of chlorine was also investigated. Using 1,2-dichlorobenzene as solvent for ferrocene in the LSCVD method, a significantly smaller wall thickness for iron-filled [41,48] and iron-carbide-filled CNT [44] was obtained in comparison with material grown by the SSCVD method [35,47]. By studying different chlorinated hydrocarbons (benzene derivatives) it was suggested that the etching effect of chlorine, formed during the thermal decomposition of the solvent, is decisive for limiting the number of carbon shells [41,48]. On the other side it could also be possible that the chlorine reacts with hydrogen to form hydrochloric acid. Simultaneously the thermally stable benzene is forming. The benzene does not intensively react in the reaction zone due to its high thermal stability. Thus the low concentration of active carbon leads to relatively few carbon shells.

The structure of the filling also depends on the process temperature and the cooling rate. At high temperatures a large amount of carbon dissolves in the catalyst particle. As Ding *et al.* [96] have theoretically shown, it is most likely that the particle is highly saturated with carbon during the growth. After a highly supersaturated state it will oscillate around the saturation concentration. If the reaction is terminated by cooling down the furnace structure transformations according to the phase diagram occur. The structure of the filling can be changed by specific tempering the sample at temperatures close to phase transitions. For example it has been shown that annealing at 645 *°*C can transform *γ*-Fe into *α*-Fe [8,48].

The phases of the filling depend also on the gas composition, gas velocity (retention period) and the method of the transfer of the precursor into the reaction zone. For example, Wang and Gui *et al.* [41,48] used the LSCVD method and evaporated a 1,2-dichlorobenzene/ferrocene solution, with a concentration of 60 mg ferrocene in 1 ml of the solvent. A gas flow of Ar/H${}_{2}$ with 2000 sccm was employed. Bundles of oriented CNT were obtained having mainly *α*- and *γ*-Fe filling. Employing the same precursor solution (same concentration) lead to mainly iron carbide filling when the solution was sprayed into the furnace with a nozzle at a transport gas flow of 1,000 sccm Ar/H${}_{2}$ [44]. However, the structures of the shells were quite similar and a significant limitation of the wall thickness was observed in both cases. If the gas flow alone is responsible for these difference can not be answered yet. The CVD-process is kinetically determined, therefore the gas velocity is decisive for the deposition process. Because the gas velocity is strongly dependent on the geometry of the used reactor (diameter, length, volume) also the deposition process is influenced strongly by the reactor geometry [51].

From the discussion above we can conclude some rules for the formation of in-situ filled CNT with different filling degree. First, for the synthesis of long and continuous filled carbon nanotubes it is convenient to use a precursor with a low carbon to metal ratio, such as it exists in the metal organic compound family of metallocenes. A metal catalyst layer as a secondary source for the filling material leads to higher filling degrees and a lower diameter distribution of the CNT. For partially filled CNT we need a higher carbon to metal (catalyst) ratio, realized by additional hydrocarbons in the precursor. A significant higher concentration of active carbon in relation to the metal catalyst will lead to less filled CNT. Still, since an additional catalyst layer leads to a smaller diameter distribution it should be considered.

For highly crystalline carbon shells rather high temperatures are advantageous. However to avoid an increase in the wall thickness the amount of transport gas should be increased. That reduced the stationary concentration of the reactive precursor and balances the amount of active carbon.

Due to the competitive reactions in the gas phase and on the substrate an optimum for the temperature has to be found for each used precursor. A reactive gas species actively takes part in the reactions. The gas velocity is important since it defines the retention period of the reacting species in the reaction zone.

### 2.5. Growth mechanism

Understanding the growth mechanism of carbon nanotubes is a key requirement for the deliberated synthesis in order to obtain the special properties needed for their applications. Intensive studies were performed to find a mechanism describing the formation of carbon nanotubes. Up to now there is no generally accepted growth mechanism and further research is necessary.

The mechanism discussed here for the formation of in-situ filled CNT is a phenomenological description. It is deduced from different experimental observations to explain the variety of observed structures of the CNT. The phenomenological mechanism is the foundation for theoretical studies. These theoretical studies are mainly performed for the formation of single-walled CNT. However, many results are also useful for the understanding of multi-walled CNT formation.

The knowledge and understanding of the mechanism is under continuing development and several modifications were discussed during the last two decades. Below selected features of the mechanism will be discussed that are especially suited for the synthesis of filled CNT by the CVD method.

#### 2.5.1. VLS mechanism

The basis of all is the vapor-liquid-solid (VLS) mechanism which was originally developed for the growth of silicon whiskers by Wagner and Ellis [97]. It consists of the assumption that a gaseous phase, a liquid phase and a solid interact. The precursor is supplied as gas. The liquid phase is the molten catalyst particle situated on the solid substrate. During the growth process the precursor decomposes in a specific area of the surface of the liquid particle at the temperature T${}_{1}$. The precursor material dissolves until saturation at concentration c${}_{1}$ is reached. It is assumed that the particle possesses an area with a lower temperature T${}_{2}$ and a lower concentration c${}_{2}$. Thus the presence of both a concentration and thermal gradient is assumed. The precursor material diffuses through the particle and precipitates at T${}_{2}$. These very basic assumptions of the mechanism allow the application to other systems.

#### 2.5.2. Base and tip growth mode

One of the first mechanism to explain the growth of carbon whiskers was initially developed by Baker *et al.* [98,99] on basis of the VLS mechanism. They performed in-situ studies on the growth of carbon whiskers employing controlled atmosphere electron microscopy (CAEM). During the formation the (liquid) metal catalyst particles were found either on the tip or bottom of the formed whiskers [99]. Due to forces acting on the catalyst particles they deform during the process resulting in two growth modes. If there is a strong attractive interaction between the catalyst and the substrate a good wettability is found that lead to contact angles below 90 *°* and the particle most likely remains on the substrate surface. Since the decomposition of hydrocarbons relies on the catalytical activity of the particle surface supplied carbon atoms can only connect to the already existing carbon structure at the particle whisker interface. Therefore the oldest part of the whisker is the tip and the youngest part is at the bottom. For this reason it is called base growth mode. If there is a repulsive interaction between the particle and the surface a contact angle above 90 *°* will establish, the particle most likely detaches from the surface and lifts up. In this case the oldest part of the CNT is close to the substrate surface and the youngest part is the tip. Thus, this is called tip growth mode. Both growth modes were introduced to discuss the different occurrence of the metal particles in the as-grown carbon material.

**Figure 9:**
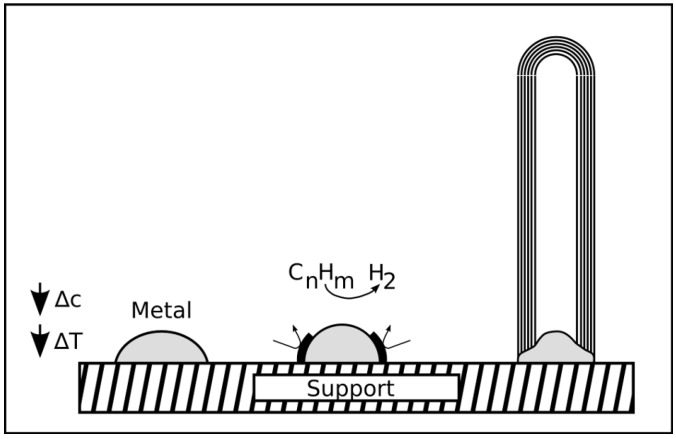
Base growth mode: The metal particles on the surface are exposed to gaseous hydrocarbons, which decompose catalytically on the surface of the catalyst particle. An exothermic decomposition is assumed and a carbon concentration as well as a temperature gradient form [99]. After its decomposition the carbon diffuses from the hot area with a higher concentration to the colder region of the particle and precipitates to form the graphitic structure of the CNT wall. The particle remains attached to the substrate.

**Figure 10:**
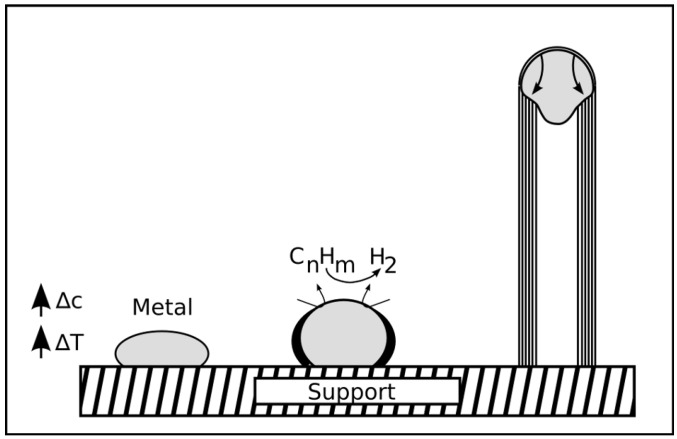
Tip growth mode: The metal particle is only weakly bound to the substrate surface. The decomposition of the hydrocarbons takes place at the upper side of the particle. Again an exothermic decomposition is assumed and the temperature and carbon concentration increases at the top of the particle which gets deformed during this process and detaches from the substrate. The carbon now diffuses to the colder side of the particle and precipitates to form the CNT shells.

Other groups adapted the mechanism of Baker *et al.* to explain their experimental results for unfilled carbon nanotubes [73]. In the modified mechanism it is assumed, that first of all the change in the size of the catalyst particles, from large dimensions down to diameters below 100 nm is the reason for the formation of the tubular carbon structures [73] instead of whiskers. The key features of the VLS mechanism like the decomposition, dissolving and diffusion of carbon species in the metal particle and precipitation due to supersaturation have been adopted and can be applied to describe the formation of carbon nanotubes. The base and tip growth mode of unfilled carbon nanotubes is shown in Figure 9 and Figure 10 respectively.

In the VLS mechanism a sufficient solubility and sufficient diffusion rate of carbon in the catalyst material is assumed. Esconjauregui *et al.* [77] investigated the catalytic effectiveness of various metals depending on their electron configuration in detail. Elements with few d-vacancies such as iron, cobalt and nickel were confirmed to be very active catalysts since they possess a high diffusion rate for carbon and form only metastable carbides. The decomposition of carbides is required for growing CNT.

The estimated growth rates suggested that the formation most often depends on the diffusion of carbon through the volume of the catalyst particle and its precipitation at an opposed side of the particle [85]. Other groups stated that only surface or subsurface diffusion takes place [100].

The dimension of the catalyst particle defines the inner diameter of the CNT but to some extent also the number of graphite shells. Sinnott *et al.* [73] investigated the influence of the catalyst particle size, the precursor composition and the temperature. They observed that the carbon shells precipitate from one half of the particle if its shape is spherical or pear-shaped (on the lower curvature face for pear shapes). As the main driving force a carbon concentration gradient was assumed [73].

#### 2.5.3. Base and tip growth mode for filled CNT

For filled CNT, not only the formation of the carbon shells but also the formation of the in-situ filling has to be explained. It is particularly difficult to describe the influence of the catalyst because it is applied on the substrate and additionally as floating catalyst from the decomposed precursor. During the whole growth process metal particles, most often catalytically active, interact with the forming CNT. Nevertheless, several groups adjusted the VLS mechanism and the concepts of the tip and base growth mode to explain the formation of *in-situ* filled CNT.

**Figure 11:**
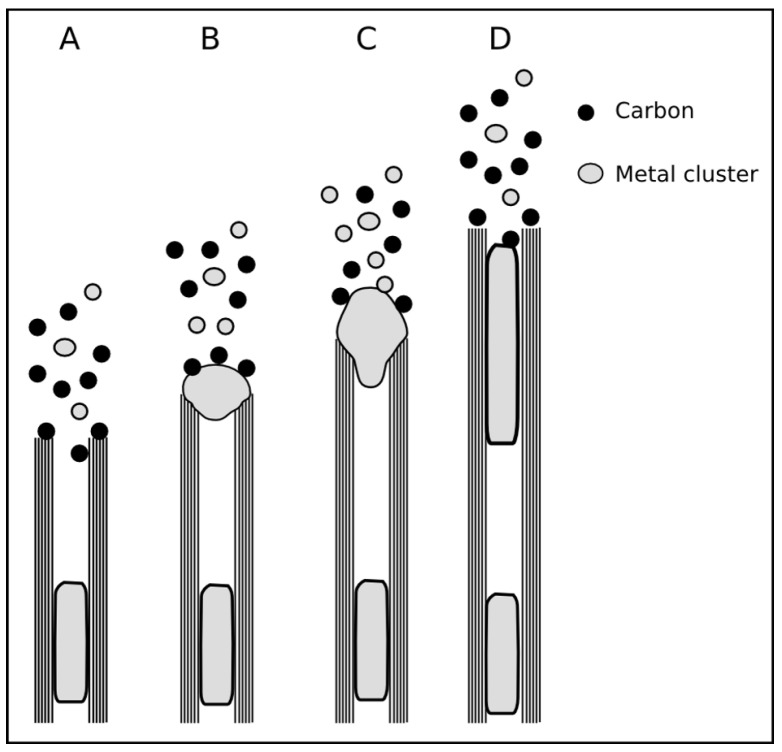
The figure presents the growth process of *in-situ* filled CNT after [38]. (A) shows the slow growth stage. The carbon shells at the open tip react with carbon clusters from the gas phase. In (B) a larger catalyst particle attaches to the open tip and the fast growth stage starts. The CNT grows fast and the pressure caused by the shells deforms the catalyst particle. In this stage (C) a filling section is formed. If the supply with catalyst material stops the slow growth stage continues.

From their experiments Zhang *et al.* [38] suggested a growth mechanism that could explain the *in-situ* filling of carbon nanotubes with iron during the synthesis. The scheme of the mechanism is shown in Figure 11.

**Figure 12:**
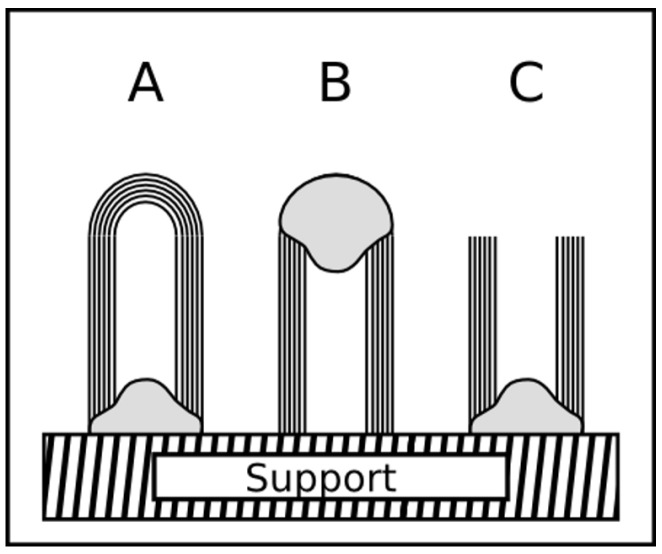
The figure shows three schematic cases for the tip of a growing CNT. Case (A) shows a closed tip since each shell is closed by a fullerene-like cap. Case (C) represents an open tip whereas this situation is only favorable under certain conditions. An open tip can be either stabilized by lip-lip-interaction [101] or as result of the so called “scooter” mechanism [102]. The case (B) is sometimes called open [103] because of the missing fullerene cap but it is also regarded as closed due to the particle.

An open-tip tip growth mode and furthermore the existence of two different growth rates were discussed. In the initial state the catalyst particles detach from the substrate surface, which results in a tip growth mode. Thereafter the growth slows down and stops eventually. An empty tube growth is explained by the reaction of carbon clusters from the gas phase with the open tip of the forming CNT. This is the slow growth stage. When a metal particle falls onto the tip the catalytic process takes place and the CNT walls grow much faster until the particle is surrounded with carbon again. This part is the fast growth stage. Both stages alternate with each other during the process. During the fast growth stage the iron particles are forced into cylindrical shape and form the filling. The pressure results from the comparatively faster carbon shell formation. The filling progresses by subsequent addition of iron nanoparticles on the open tip of the growing CNT. However, the assumption of an open tip during the growth is critical. There are differing possibilities to define an open tip and two cases are shown in Figure 12(B),(C).

Since the existence of open tips is a fundamental assumption in some of the growth mechanisms [38,43] for filled CNT, we want to briefly discuss the possibility for their occurrence during the CNT formation. Most of the discussion in literature is related to unfilled and especially single-walled CNT. However, the understanding of these investigations can be transferred to some extent to the carbon shell formation of filled CNT.

Kwon *et al.* [101] simulated the growth of unfilled MWCNT without a metal catalyst involved. The basic idea is that dangling bonds at the open tip are stabilized by single atoms that form stable covalent bridge-bonds between adjacent layers which are called lip-lip interactions. The CNT grows by subsequent incorporation of carbon atoms into the shell structure. The formation of a cap requires more energy than the lip-lip interactions and excess carbon is needed for building pentagonal defects. This distinguishes the mechanism from others, because cap formation, in the lip-lip interaction mechanism interrupts and finishes the growth process. However, another type of simulation revealed, that the lip-lip interaction is not sufficient to keep the tip of a growing CNT open [104,105]. In other mechanisms the formation of a cap is the initial step and often called nucleation of the CNT growth process [103].

An alternative mechanism to prevent the closure of the growing CNT is the “scooter“-motion. A fast moving small metal cluster on the open tip prevents the closure of the CNT cap [102]. In the CVD synthesis of *in-situ* filled CNT there is a relatively high concentration of small metal clusters in the gas phase [88] and the tip of a growing CNT interacts with these clusters. It is hardly possible that the lip-lip or scooter mechanism are stable under these conditions. It is therefore assumed that either the tip of the CNT is closed by a carbon cap or by a metal particle with a diameter at least of the inner diameter of the CNT [103]. In case of MWCNT the particle diameter should be as large as the outer diameter (see Figure 12(B)). Also Charlier *et al.* [106] found that a metal particle under their simulation conditions promotes a closure of the cap. Most of these simulations are restricted to the formation of single-walled CNT, but are also valid for multi-walled CNT.

An experimental evidence for an open tip growth was reported by Iijima *et al.* [72] whereas the material was synthesized by arc discharge. It was suggested that the continuous growth of long CNT results from addition of hexagons whereas heptagons together with pentagons lead to the formation of closed caps. The thickness of the CNT increases by subsequent addition of further carbon on existing basal planes. An open tip growth was also proposed to explain the experimental data [37,38,43,85]. Open caps are not exclusively observed by transmission electron microscopy (TEM).

**Figure 13:**
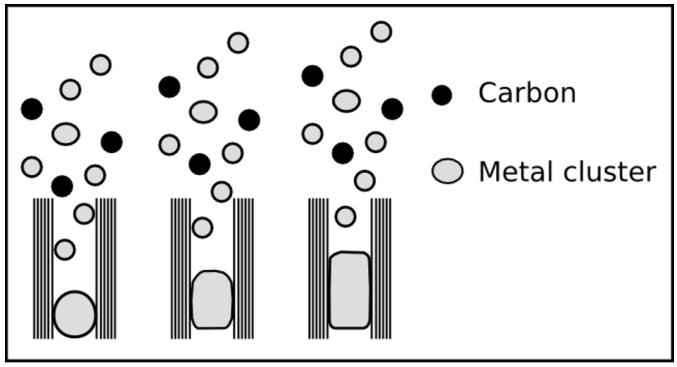
Open tip base growth: The filling of the cavity results from the diffusion of small metal clusters that are produced in the gas phase. The clusters diffuse a certain distance, eventually forming a continuous filling.

According to Deck *et al.* [43] the formation of iron-filled carbon nanotubes can be explained by the open tip base growth mechanism which is shown in Figure 13.

The iron-filled CNT were produced by the spray pyrolysis method. It is suggested that the long iron nanowires inside of the CNT occur through the subsequent addition of iron nanoclusters that fall onto the open tip. The liquid-like metal clusters with diameters lower than the inner diameter of the CNT diffuse inside the cavity. They can contribute to an existing nanorod or form a new one. For the diffusion to take place, a liquid or at least highly mobile state of the clusters is required. In this work [43] no slow and fast growth stage is assumed. However, the mechanism offers an explanation for micrometer long metallic wires encapsulated in carbon nanotubes. Deck *et al.* also proposed the formation of small CNT embryos in the gas phase that fall onto growing CNT.

The presented mechanisms are capable of describing the formation of both the filling and the carbon shells in a feasible manner. However, theoretical studies [96,103,104,105,106,107] have shown, that an open tip in the sense of Figure 12(C) is energetically unfavorable in comparison to a closed cap.

#### 2.5.4. Combined growth mode

An intensive discussion and interesting approach to explain the growth of metal-filled CNT was presented by Kunadian *et al.* [108]. They combined the tip- and base-growth mode explaining the formation of the filling in the context of a closed tip growth process (compare Figure 12(B)). In the continuous-feed CVD the precursor containing both the carbon source and the catalyst material is provided continuously during the process.

**Figure 14:**
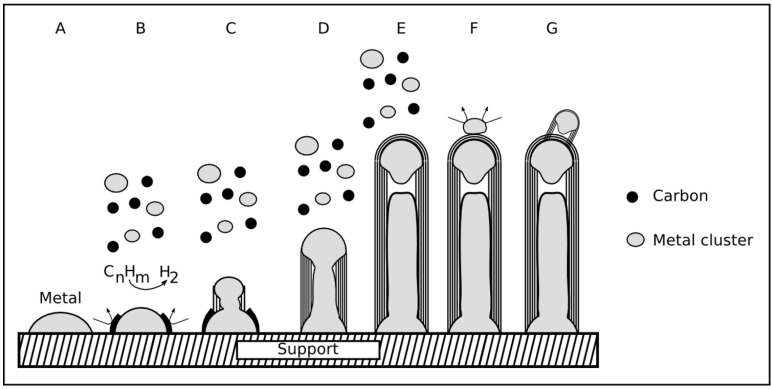
Steps of the combined growth mechanism. In step A a catalyst particle is formed. Step B describes the decomposition of hydrocarbons on the particle surface. Due to the floating catalyst method catalytic processes occur also in the gas phase forming metal and carbon clusters. In step C the deposition of iron particles at the growing site takes place continuously, thereby the growth mode changes from base to tip growth mode (step C→D). When further material deposits from the gas phase the growth continues (step E) until a stable cap is formed. If the cap is closed so called secondary growth might occur. This is often tip growth since the wettability of the metal catalyst is low and the particles easily detach (step F and G). According to [108]

In the mechanism (Figure 14) it is assumed that the initial state follows the base growth mode. Hydrocarbons decompose on the surface of the catalyst particles. Due to the floating catalyst conditions metal clusters are formed and hydrocarbons can also decompose already in the gas phase. The catalytically active metal clusters and the carbon atoms fall onto the catalyst particles which remain anchored to the substrate surface. One may assume, that this results in a continuing change of the catalyst conditions. Each cluster that connects to the catalyst particle modifies the volume, shape and the carbon concentration of the catalyst. Thus under floating catalyst conditions it is likely that the growth mode switches from the base to tip growth mode. After the switching the filling process as well as the shell formation occurs simultaneously at the tip.

The mechanism can thereby explain many observed phenomena like the distribution of metal particles and wires along the CNT hollow core. Furthermore, structure specifics such as kinks and branches of CNT that are characteristic for filled CNT are included.

As mentioned the closed tip growth (see Figure12(A),(B)) is an important assumption in this mechanism. After the CNT have stopped to grow a secondary nucleation can take place and further CNT are formed on the existing material. These new CNT are generally formed by the tip growth mode since the interaction between the metal particles with the already existing CNT material is low and particles detach [108]. The combined mode proposed can explain many experimental observations with only making a few assumptions, and taking theoretical knowledge into account.

Experimental results of the *in-situ* synthesis of metal-filled CNT [35,67] support the idea of the combined growth mechanism. In the experiment different materials for the substrate catalyst and the floating catalyst were selected. At first, a Fe catalyst layer (2 nm) and cobaltocene as precursor was employed as carbon and metal source. Energy dispersive X-ray spectroscopy (EDX) investigation in the middle of the filling shows resolvable amounts of iron [67]. Secondly, a Co layer (2nm) on a silicon substrate was employed and ferrocene was used as carbon and metal source. Vertically aligned iron-filled CNT could be achieved. Investigations by cross sectional EDX inside TEM revealed that there is no Co at volume sections along the Fe filling [67,109]. The latter is an indication for switching from base to tip growth mode since only the material from gas phase (Fe) was catalytically active and found as filling phase.

#### 2.5.5. Conclusion

The number of individual growth mechanisms that differ partially or completely in their assumptions and explanations shows that the present knowledge is not sufficient to completely describe the formation of *in-situ* filled carbon nanotubes by chemical vapor deposition. The properties of carbon nanotubes are strongly influenced by the reaction conditions and different mechanisms may be involved in their formation. The reaction conditions determine the influence of each mechanism involved in the formation process. The phenomenological mechanisms are developed on the basis of experiments and thus are influenced by the corresponding process conditions. The comparison of results from different groups is often complicated due to the differing process parameters and growth conditions. A coherent growth mechanism should cope with all experimental results.

## 3. Properties

### 3.1. Structure

In this section an overview about the structural properties of metal-filled CNT is presented. In the first part, the structure of the magnetic filling will be presented and the outstanding magnetic properties of encapsulated ferromagnetic nanowires will be discussed in the next section.

**Figure 15:**
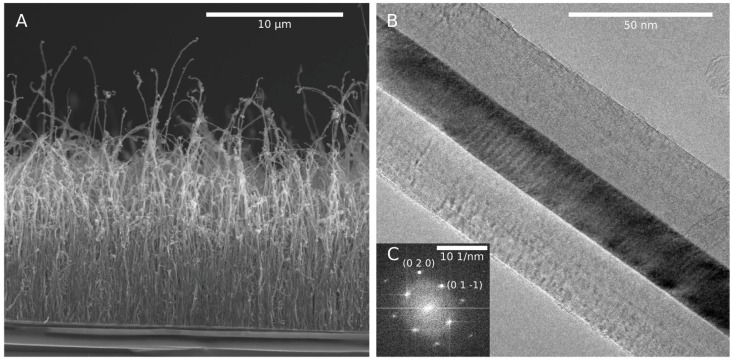
(A) Shows a SEM of an as grown sample of iron-filled CNT. In (B) a TEM micrograph of the filling and the carbon shells is presented. In (C) the corresponding diffraction pattern of the iron filling is visible.

As the ferromagnetic wires are encapsulated inside the cavities of the CNT the quality of the shell structure is an important characteristics. A first impression of the as-grown material can be obtained by scanning electron microscopy (SEM) and often shows important features. A characteristic SEM image of iron-filled CNT grown via SSCVD method is shown in Figure 15(A).

**Figure 16:**
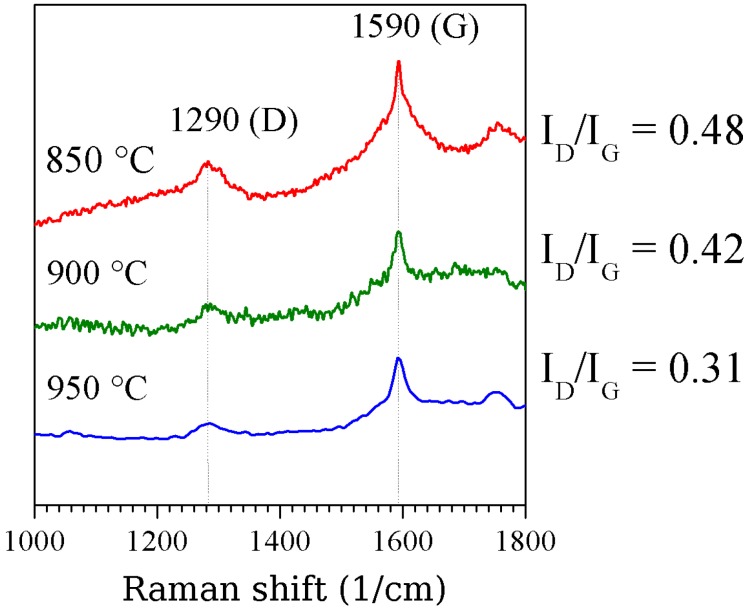
Raman spectra of Fe-CNT [12]. The crystallinity of the CNT structure increases with increasing reaction temperature. The intensity of the D-band decreases relatively to the intensity of the G-band.

Structural features such as diameter and length distribution, alignment of the CNT and formation of spherical particles can be determined. For studies of structural details the CNT are investigated by transmission electron microscopy. A high resolution image together with the corresponding diffraction pattern are shown in Figure 15(B),(C). Information about the crystallinity of bulk material can be measured by Raman spectroscopy. Raman spectra of three different samples grown at three different temperatures are shown in Figure 16. For CNT, the active tangential mode (G-line) is found around 1600 cm${}^{-1}$, which results from the E${}_{2g}$-mode of graphite. In the case of a perfect crystalline structure only this line would be found. However, the defect-induced mode (D-line) around 1300 cm${}^{-1}$ is always found. This line results from a Raman double scattering process and is proportional to the defect density in the CNT.

The floating catalyst growth conditions that lead to the in-situ filling influence the structure of the shell. If long and continuous nanowires are requested a shell closure due to cap formation and interruption of the growth should be avoided. The floating catalyst conditions increase the probability for certain defects such as branching and formation of spherical inclusions. Often also larger spherical particles are deposited on the grown CNT.

In this section we summarize the current knowledge about the structural properties of the filling of carbon nanotubes synthesized by the both in-situ methods SSCVD and LSCVD.

**Figure 17:**
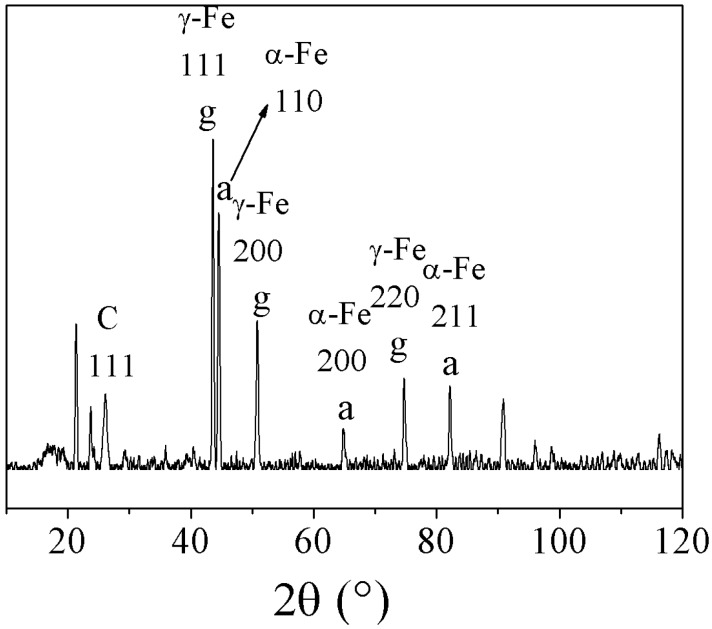
X-Ray diffraction spectra of Fe-CNT. The *α*-Fe and *γ*-Fe are found [12].

Especially iron-filled carbon nanotubes are intensively investigated. Considering the known binary phase diagram of iron and carbon, we can assume that by the in-situ filling and the used CVD parameters (especially the synthesis temperature) in principle three different phases are possible inside the nanotube, like in the bulk: the ferromagnetic *α*-iron, the paramagnetic *γ*-iron or/and the ferromagnetic metastable iron carbide. In order to obtain information about the composition and structure of the filling material different methods are employed. In general X-ray diffraction (XRD) techniques can be used to prove the different modifications, e.g., in the Brentano-configuration [35] or XRD with glancing incidence of the X-ray radiation at different angles. The latter allows the measurement of phase profiles along the vertical direction of aligned CNT deposited on a substrate [8]. Near the substrate mainly *α*-Fe and Fe${}_{3}$C was found, however *γ*-Fe was found near the tips of filled carbon nanotubes [8,35,86]. But the XRD does not often give a satisfying quantitative characterization of the phase composition. In Figure 17 a X-ray diffraction spectra of iron-filled CNT is shown.

**Figure 18:**
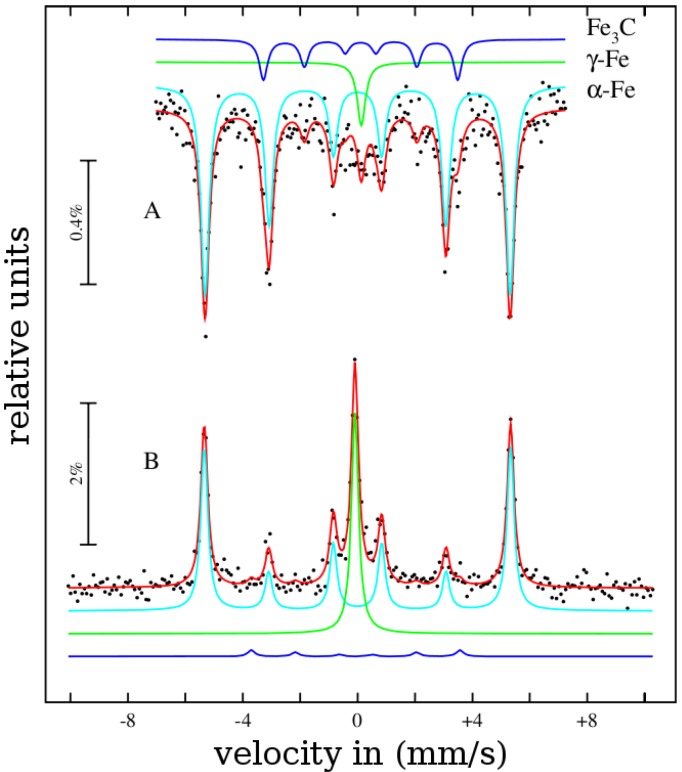
Typical Mössbauer spectra of aligned iron-filled CNT on a substrate. Spectra A results from TMS and spectra B from CEMS.

Another convenient method for the investigation of the filling material is the ${}^{57}$Fe Mössbauer spectroscopy [110], that was applied both as transmission Mössbauer spectroscopy (TMS) and

backscattered conversion electron Mössbauer spectroscopy (CEMS) to distinguish the different Fe phases and their spatial distribution within the whole sample and along the tube axis. The Mössbauer spectroscopy can be performed on CNT powder as well as on aligned CNT samples deposited on different substrates. A Mössbauer spectra of iron-filled CNT is presented in Figure 18.

XRD and Mössbauer spectroscopy together with imaging and analytical scanning and transmission electron microscopy are the typical methods for the structural characterization of filled carbon nanotubes.

Electron energy loss spectroscopy (EELS) line scans across individual tubes performed by Grobert *et al.* [111] indicate supplementary the pure iron, anti correlated to carbon and no other elements as oxygen or sulfur. Selected area electron diffraction (SAED) in a TEM or FFT of HRTEM images can disclose the different modifications of the metals iron [112,113,114], Co [115] and Ni [116]. Co is detected inside the tubes as fcc *α*-Co although this phase is only stable above 450 *°*C (in bulk) and the diffraction is realized at room temperature. It seems that the relatively inflexible carbon nanotube prevents a phase transformation and stabilizes the *α*-Co. Iron fillings present a similar phenomenon. The high temperature phase *γ*-Fe (in bulk stable higher as 1013 K) is also observed as filling material at room temperature, the nanotube stabilizes also this phase. Depending on the growth conditions, at Fe-filled carbon nanotubes besides *α*- and *γ*-Fe also significant amounts of iron carbide was found by different authors [41,43,112].

As mentioned above, the Mössbauer spectroscopy can show additionally the local distribution of the different phases along the axis of tubes. The relative content of *γ*-Fe decreases from the tip to the root of tube (or from top to the bottom of carpet on a substrate) whereas the relative content of iron carbide increases [117]. At the interface CNT/substrate mainly *α*-Fe and Fe${}_{3}$C were found. An open question is the disagreement between the results of Ruskov *et al.* [117] and other authors [110,118]. Whereas Ruskov only found single phase particles, *α*-, *γ*-Fe and Fe${}_{3}$C respectively, Marco *et al.* [110] and Prados *et al.* [118] suggested that also Fe-filled MWCNT consist of an *α*-Fe particle core, surrounded by the shell of *γ*-Fe phase and finally covered by a layer of Fe${}_{3}$C, which is located at the interface between the metallic phases and the inner wall of carbon nanotubes. Golberg *et al.* [113] could also detect by HRTEM that iron carbide is localized near the graphitic shells, whereas the filling core is *α*-Fe. But always between Fe${}_{3}$C and carbon shell, a rather thin layer of *α*-Fe is observed due to the metastability of the interface C/Fe${}_{3}$C (Fe${}_{3}$C + C → 3 Fe + 2C). By TMS investigation it was also found that depending on the synthesis conditions, the distribution and the filling degree of Fe in aligned grown tube carpets could be different, but the ratio *γ*-Fe/*α*-Fe is relatively constant. A combining of TMS and CEMS and a comparison of both spectra confirms again the tendency that the iron carbide is more localized near the substrate and is absent directly on the surface of the carpets. Such information about the phase distributions could be important for the understanding of the growth mechanism.

It is worth to mention that the paramagnetic *γ*-Fe of an iron nanowire limits its use for magnetic applications. A complete transformation into *α*-Fe is necessary. By an annealing process scarcely below the transformation temperature *γ* →*α* the entire paramagnetic iron can be transformed into ferromagnetic and thus an increase of the magnetic moment of the sample is observed [86].

### 3.2. Magnetic properties

**Figure 19:**
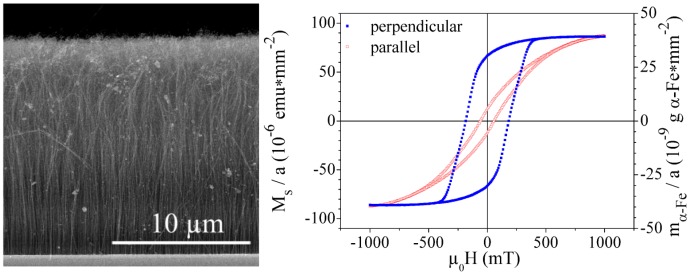
SEM and AGM of a bulk sample [12]. A large hysteresis and anisotropy can be observed.

As a first approach to explore the magnetic behavior of carbon nanotubes filled with ferromagnetic materials, many groups applied bulk magnetometry, e.g., alternating gradient magnetometry (AGM) to as-grown nanotube samples [8,12,86,111,115,119]. In Figure 19 the cross section of a bulk sample and the corresponding hysteresis curves are shown.

Basic results provided by these measurements include the confirmation of ferromagnetism and a strong magnetic anisotropy (in most cases dominated by the nanowire’s shape). Room temperature coercivities of ensembles of Fe-filled CNT up to 180 mT have been reported [12]. The saturation moment m${}_{s}$ allows for an estimation of the total volume V or mass of the ferromagnetic material involved [12].

(2) $$V\;=\;\frac{m_{s}}{M_{s}}$$

Here, the saturation magnetization $M_{s}$ needs to be known, e.g., bulk $M_{s}$ values might be used. In general, the data evaluation of CNT ensemble magnetometry needs to be done with care. Depending on the CNT packing density and the degree of filling with ferromagnetic materials, dipolar interactions have to be taken into account. In case of samples with non-interacting nanowires, magnetometry measures the hysteresis loops of many individual nanowires, averaged or integrated over a broad distribution of nanowire lengths, diameters, shapes and deviations from parallel alignment. Magnetic measurements of individual CNT avoid such problems. Magnetic force microscopy [44,46,120] and electron holography [115,121,122,123,124] have been applied to image stray field gradients and magnetic fluxes generated by single filled CNT. The magnetic moments of individual filled CNT, their switching and anisotropy fields have been measured by cantilever magnetometry [125].

The remanent state of ferromagnetic nanowires inside CNT is most often a single domain configuration which is expected for the considered nanowires size [126]. In the case of Fe nanowires the remanent magnetization is aligned along the long nanowire axis, *i.e.*, the magnetic anisotropy is dominated by the shape [46,120,121,125]. High aspect ratio Fe nanowires can be regarded as consisting of two well separated magnetic monopoles located near the two wire ends. In contrast, CNT filled with Fe${}_{3}$C reveal a magnetization perpendicular to the CNT axis that can be explained in terms of a strong magnetocrystalline anisotropy. Switching fields of single Fe nanowires inside CNT between 100 and 400 mT have been reported [46,120,125]. Reports on CNT contained Fe nanowires reveal magnetization values close to the material’s bulk magnetization [122,125,127] and in case of Co nanowires a reduced magnetization [123]. In most reports, the magnetic response of the filled CNT is attributed solely to ferromagnetic phases of the filling. However, some authors found hysteresis shifts explained by exchange bias effects at low temperatures [118,128]. Prados *et al.* described Fe-filled CNT that show exchange bias effects at low temperatures caused by *γ*-Fe/*α*-Fe antiferromagnetic-ferromagnetic interfaces [118].

Theoretical studies concerning the magnetic properties of CNT filled with ferromagnetic materials deal, in most cases, with single walled carbon nanotubes [129,130,131,132]. In the case of Fe nanowires enclosed in SWCNT, enhanced magnetic Fe moments are predicted and explained by the low coordination number of Fe atoms in these systems [130,131]. The magnetic moments of single Fe atoms inside CNT depend on the atoms’ specific locations [133]. The magnetic moment of Fe atoms close to CNT defects is expected to be reduced [132]. The preparation and unambiguous magnetic characterization of SWCNT filled with ferromagnetic materials seems to be a challenging task, however, a couple of reports exist [134,135]. Jorge *et al.* describe room temperature ferromagnetic behavior in the case of Fe-filled double wall carbon nanotubes [136].

## 4. Applications

### 4.1. Medical Applications

The usability of nanosize magnetic particles in biomedical applications is very active field of research. In this article we focus on selected applications where CNT filled with extended magnetic nanowires might be used. Filling carbon nanotubes with tailored materials realizes nanoscaled containers in which the active content is encapsulated by a protecting carbon shell. Especially the encapsulated iron is efficiently protected by the surrounding shells and its magnetic properties are retained. One medical application of ferromagnetic material is the so-called “magnetic fluid hyperthermia” (MFH). Here the idea is that a cancer cell will be killed when a temperature above 41–42 *°*C is maintained in the target volume. The magnetic material can be localized in deep tissue by gradient fields that can move them and alternating (AC) fields lead to local heating. Much of the current research is focused on iron oxide nanoparticles which have proven their feasibility in animal experiments [137,138] and are now under clinical trials [139]. Jordan *et al.* [140] made large progress in the research and development of these nanoparticles to clinical applications in patients. The use of nanoparticles made of iron, however, is hindered by the fact that oxidation in ambient or biological conditions has to be avoided. A promising way to overcome this problem appears to be the iron-filled CNT and thereby protecting the biological environment and the filling material against each other. Degradation of the filling materials is avoided and their potential toxicity and adverse effects are suppressed so that CNT provide a smart carrier system on the nanometer scale. Klingeler *et al.* [21] published an extensive overview about iron-filled CNT in medical applications. Magnetic studies on the feasibility of iron-containing carbon nanotubes as appropriate material for magnetic hyperthermia have been shown recently [141]. Accurate control of the tissue temperature is mandatory in any hyperthermia approach. Currently, in clinical trials already available for the magnetic fluid hyperthermia therapy, temperature is controlled by a clinician’s intervention by placing thermocouples or fiberoptical thermometers into the tumor [142]. A novel contactless thermometry on the cellular level by using a nanoscaled thermometer, which consists of a carbon nanotube and a filling material with strongly temperature dependent nuclear magnetic resonance (NMR) parameters at 310–350 K, has been reported. Temperature detection can thus be made with a high accuracy (≤0.1 degree). In particular, the filling with magnetic material offers the potential for hyperthermia applications while the insertion of NMR active substances allows the usage as markers and sensors. Up to now, the more simple system has been studied, where only the temperature sensor (CuI) is encapsulated in CNT [143]. Another important field of research is the application of iron oxide coated or iron-filled SWCNT as contrast agent for magnetic resonance imaging (MRI) [144,145]. Finally, the container feature of CNT allows the filling with an additional therapeutic agent like cisplatin or carboplatin [31,146] as a potential drug delivery systems. These CNT filled with a magnetic material and a therapeutic can be transferred into cells and so the therapy can be realized at cellular level, the chemotherapy and the thermotherapy. That implies less therapeutic is needed in comparison to a conventional therapy and lower side effect for the patients can be expected. Besides CNT, encapsulating magnetic nanoparticles and nanowires might also be studied for cell separation and manipulation [147,148,149].

### 4.2. Sensor Applications

Carbon nanotubes filled with ferromagnetic materials constitute stable nanomagnets with no need for supporting structures like substrates. Filled CNT maintain constant properties without mechanical or chemical degradation. This makes them excellent components for magneto-mechanical sensors. CNT are already known to be suitable for high quality scanning force microscopy probes [150]. Magnetic force microscopy (MFM) is a technique based on scanning force microscopy. Here, the magnetic moment of the sensor interacts with the sample’s magnetic stray field. A couple of experimental studies introduce CNT based MFM sensors using, e.g., metal coated CNT [151,152], metal capped CNT [153,154,155], or metal-filled CNT [45,46,156]. Ideally, an appropriate MFM probe would consist of an elongated single-domain needle made from high remanence material [157]. These requirements can be explained as follows. A high aspect ratio supports the magnetic shape anisotropy and thus stabilizes the probe’s magnetization. The MFM signal strength is proportional to the probe’s magnetic moment or its remanent magnetization. A single domain configuration maximizes the overall magnetic moment of the probe. In a broad range of imaging conditions, a probe with an elongated cylinder shape might be regarded as a magnetic monopole of which only the monopole close to the sample surface contributes to the magnetic interaction. In the framework of Fourier transfer functions [158], a probe’s monopole behavior is equivalent to a region of a constant force transfer function. The monopole approach is attractive because it leads to a simple proportionality between the first derivative of the sample’s stray field and the MFM signal in dynamic MFM. The preparation of MFM probes using filled carbon nanotubes is still a delicate issue. There are reports on the in-situ CVD growth of metal capped carbon fibers on cantilever probes [155]. Yet the in-situ preparation of filled CNT on cantilever probes needs to be implemented. The latter has been bypassed by mechanically attaching filled CNT to cantilever probes [45,46]. Such procedures may include the following steps. First, filled CNT are grown by chemical vapor deposition on catalyst-coated silicon substrates. Selected nanotubes are then attached to conventional scanning force microscopy tips using a SEM equipped with a micromanipulator. After this, the fabricated probes are inspected and can, if necessary, be tailored by etching off unwanted carbon parts by localized electron-beam induced oxidation in an SEM. Fe-filled CNT proved to be high-resolution long-lasting MFM probes. These sensors are expected to meet the requirements of ideal MFM probes as outlined above. In particular the verification of the monopole approach will allow for an easy implementation of quantitative MFM approaches. In addition, the magnetic stability of Fe-filled CNT probes has been demonstrated by MFM measurements in in-plane fields up to 250 mT [156].

Finally we would like to present a proposal for a CNT-based magnetic switch. In the CNT MFM probe approach described above the CNT’s elastic properties are not actively used although the CNT’s flexibility is beneficial for preventing destructive probe-sample crashes. Similar to electromechanical CNT relays [159] it could be possible to fabricate magneto-electromechanical nano-switches based on CNT filled with ferromagnetic material. A one-side clamped filled CNT exposed to an external magnetic field would bend in response to the torque acting on its magnetic moment. Subsequently, the CNT would make contact with an appropriate drain electrode.

## 5. Summary

In this review we present an overview about the synthesis of CNT filled with ferromagnetic materials by thermal chemical vapor deposition. Different setups and process conditions are discussed. We have shown the influence of different growth parameters on the *in-situ* formation of filled CNT. The inner and outer diameter of CNT depend on the diameter of the catalyst particles. However, the outer diameter is also influenced by the concentration of reactive carbon species, which is controlled by the reaction temperature and the precursor species. The structure of the filling depends on the synthesis parameters such as temperature, precursor, gas atmosphere and cooling rate. Also a brief overview and discussion about the underlying phenomenological growth mechanisms is presented. There is a lack of understanding the growth process by simulation. However, simulation of the floating catalyst process might lead to a better understanding of the mechanisms involved. Ferromagnetically filled CNT possess interesting magnetic properties such as a high shape anisotropy and single domain configuration. These nanomagnets are promising material for several applications at the nanoscale. Partially filled CNT can be employed as medical drug delivery systems. Continuously filled CNT can be used as magneto-mechanical actuators in the MEMS and NEMS technology.
